# Steroidal Alkaloids from Food Waste of Tomato Processing Inhibit Neuroblastoma Cell Viability

**DOI:** 10.3390/ijms242316915

**Published:** 2023-11-29

**Authors:** Debora Santonocito, Matteo Delli Carri, Agatina Campisi, Giovanni Sposito, Rosalia Pellitteri, Giuseppina Raciti, Nunzio Cardullo, Giovanna Aquino, Manuela Giovanna Basilicata, Giacomo Pepe, Rosario Pignatello, Carmelo Puglia

**Affiliations:** 1 Department of Drug and Health Sciences, University of Catania, Viale Andrea Doria 6, 95125 Catania, Italy; campisag@unict.it (A.C.); giovanni.sposito@unict.it (G.S.); rosario.pignatello@unict.it (R.P.); capuglia@unict.it (C.P.); 2 NANOMED-Research Center on Nanomedicine and Pharmaceutical Nanotechnology, University of Catania, 95125 Catania, Italy; 3 Department of Pharmacy, University of Salerno, 84084 Fisciano, Italy; mdellicarri@unisa.it (M.D.C.); gaquino@unisa.it (G.A.); mbasilicata@unisa.it (M.G.B.); gipepe@unisa.it (G.P.); 4 Institute for Biomedical Research and Innovation (IRIB), National Research Council, Via P. Gaifami 18, 95126 Catania, Italy; rosalia.pellitteri@cnr.it; 5 Department of Chemical Sciences, University of Catania, Viale Andrea Doria 6, 95125 Catania, Italy; ncardullo@unict.it; 6 PhD Program in Drug Discovery and Development, University of Salerno, 84084 Fisciano, Italy

**Keywords:** tomato, food waste, cell viability, leaves tomato

## Abstract

Nowadays, there is considerable attention toward the use of food waste from food processing as possible sources of compounds with health properties, such as anticancer activity. An example is tomato processing, which is responsible for generating a remarkable amount of waste (leaves, peel, seeds). Therefore, our goal was to evaluate the potential anticancer property of tomato extracts, in particular “*Datterino*” tomato (DT) and “*Piccadilly*” tomato (PT), and to study their phytochemical composition. Liquid chromatography with tandem mass spectrometry (LC/MS-MS) results showed that these extracts are rich in alkaloids, flavonoids, fatty acids, lipids, and terpenes. Furthermore, their potential anticancer activity was evaluated in vitro by MTT assay. In particular, the percentage of cell viability was assessed in olfactory ensheathing cells (OECs), a particular glial cell type of the olfactory system, and in SH-SY5Y, a neuroblastoma cell line. All extracts (aqueous and ethanolic) did not lead to any significant change in the percentage of cell viability on OECs when compared with the control. Instead, in SH-SY5Y we observed a significant decrease in the percentage of cell viability, confirming their potential anticancer activity; this was more evident for the ethanolic extracts. In conclusion, tomato leaves extracts could be regarded as a valuable source of bioactive compounds, suitable for various applications in the food, nutraceutical, and pharmaceutical fields.

## 1. Introduction

Tomato (*Solanum lycopersicum* L.) is the most consumed vegetable worldwide [1]. It originated from Latin America, and subsequently its cultivation and consummation spread throughout the world [2]. It is consumed as fruit (salads, cold dishes) or processed into tomato-based products (sauces, ketchup).

Tomato processing is responsible for generating a remarkable amount of food waste. During the harvesting, the tomato plants (stems, peel) are discarded and used for feeding livestock without any economic benefit for the producers, while the tomato fruits are harvested. Substantial quantities of these fruits are processed into sauces and juices, generating a significant amount of waste (seeds, leaves, peel) [3,4]. As reported in the literature, all these natural by-products are rich of bioactive compounds (alkaloids, flavonoids, phenols, carotenoids), conferring them many health properties, such as antimicrobial, antiviral, and antioxidant activity [5,6,7,8,9,10]. Among the best-known bioactive compounds are carotenoids (lycopene, β-carotene and lutein), which are able to reduce the risk of degenerative disorders, such as macular degeneration, cancer, and cardiovascular diseases, due to their antioxidant properties [4]. Currently, two steroidal alkaloids, tomatine (TM) and tomatidine (TD), are attracting considerable attention due to their potential health benefits. Both molecules are mainly contained in tomato by-products (leaves, roots, and tomato peel) and show many beneficial properties such as antioxidant, antimicrobial, anti-inflammatory, and anticancer activities [11].

In order to design more sustainable production chains and recover food waste, the theory of circular economy (CE) has been promoted as a promising strategy to reduce the amount of waste and exploit its beneficial properties. Silva-Beltran and coworkers demonstrated the antioxidant and antimicrobial activities of tomato leaves extracts from two tomato Mexican cultivars (*Pitenza* and *Floradade*) [3]. In particular, the *Pitenza* variety exhibited the highest antioxidant and antimicrobial activities due to the high content of tomatine, chlorophyll, and flavonoids. In another work, the antimicrobial activity of tomato leaves, stems, and fruit was studied, highlighting the remarkable efficacy of the leaves extract due to the high tomatine content [12]. Moreover, Fujimaki and his research group demonstrated that tomatidine-rich tomato leaf extract (TRTLE) was able to significantly inhibit tumor growth in vivo and the proliferation of human gastric cancer cells in vitro [13].

Based on these promising results, the aim of this study was to investigate the leaves’ phytochemical composition of two Protected Geographical Indication [14] (PGI, IGP) Sicilian tomato varieties (“Datterino” tomato, DT, and “Piccadilly” tomato, PT; *Lycopersicon lycopersicum* and *Lycopersicon Esculentum*, respectively), in order to exploit and allow “a second life” to these precious by-products. All extracts were characterized by ultra-high-performance liquid chromatography coupled to high-resolution mass spectrometry (UHPLC-HRMS/MS). Moreover, their potential anticancer activity was evaluated in vitro by MTT assay. In particular, the goal was to assess the percentage of cell viability of OECs, a particular glial cell type of the olfactory system showing stem cell characteristics, and on SH-SY5Y, a neuroblastoma cell line, exposed to different concentrations of the obtained extracts.

## 2. Results and Discussion

### 2.1. Characterization of Extracts

Liquid chromatography with tandem mass spectrometry analysis (LC/MS-MS) was carried out on DT and PT tomato extracts (aqueous extracts: DTA and PTA; first ethanolic extracts: DTE1 and PTE1; second ethanolic extracts: DTE2 and PTE2), enabling the identification of numerous compounds, primarily belonging to the glycoalkaloid and fatty acid classes (Figures S1 and S2). The comprehensive list of tentatively identified compounds in tomato extracts is reported in Table 1.

The chromatogram of each sample analyzed in negative ionization mode showed a peak 1 with an [M+CH_2_O_2_-H]^−^ ion at *m*/*z* 431, along with a fragment ion at *m*/*z* 385 representing the loss of formate [M−H-46]^−^. The fragment ion at *m*/*z* 233 is generated by the removal of a sugar moiety [M−H-46-162]^−^. This peak has been attributed to roseoside [15].

Rutin (peak 2) consistently showed an identical fragmentation pattern irrespective of the ionization mode: this flavonoid showcased [M−H]^−^ at *m*/*z* 609 and [M+H]^+^ at *m*/*z* 611. Notably, in both analytical modes, the primary fragment corresponded to the aglycone at *m*/*z* 301 (in the case of negative ionization mode) and at *m*/*z* 303 (in the case of positive ionization mode) [16].

Azelaic acid (peak 6) was associated with the deprotonated molecule at *m*/*z* 187 [M−H]^−^, which generated the fragment ions at *m*/*z*: 169 [M−H-H_2_O]^−^, 143 [M−H-CO_2_]^−^, and 125 [M−H-CO_2_-H_2_O]^−^ [17].

A similar fragmentation pattern can also be observed for *m*/*z* 243 [M−H]^−^. 4-oxo-dodecanedioic acid (peak 7) yielded fragment ions at *m*/*z*: 225 [M−H-H_2_O]^−^, 199 [M−H-CO_2_]^−^, and 181 [M−H-CO_2_-H_2_O]^−^ [18].

Based on its negative fragmentation pattern, peak 19 was tentatively identified as 16-hydroxy-9-oxo-10E,12E,14E-octadecatrienoic acid. A series of cleavages was observed within the conjugated triene structure (C_10_–C_11_ and C_15_–C_16_), yielding corresponding peaks at *m*/*z* 235 [M-C_4_H_7_O-H]^−^, *m*/*z* 185 [M-C4H_7_O-H]^−^, and *m*/*z* 121 [M-C_10_H_17_O-H]^−^ [19].

Peak 14 exhibited a molecular ion at *m*/*z* 327 [M−H]^−^, corresponding to the molecular formula C_18_H_32_O_5_. The deprotonated molecular ion then yielded a series of ions at *m*/*z* 229 [M−H-C_6_H_10_O]^−^ and *m*/*z* 211 [M−H-C_6_H_10_O-H_2_O]^−^. According to the scientific literature, this fragmentation pattern was tentatively identified as malyngic acid [20,21].

Under a negative ionization mode, multiple pairs of fatty acids linked together were detected, with only one specific pair also linked to a glucoside unit. Peak 23 was tentatively identified as palmitoleic-linolenic glucoside in each chromatogram analyzed using a negative ionization mode, except for samples subjected to aqueous extraction. The deprotonated molecule at *m*/*z* 721 [M−H]^−^ produced as a main fragment *m*/*z* 277, which was assigned to the C_18_H_32_O_2_^−^ ion corresponding to deprotonated linolenic acid.

A similar fragmentation pattern was also observed for ions at *m*/*z* 559 [M−H]^−^, *m*/*z* 561 [M−H]^−^, and *m*/*z* 537 [M−H]^−^, which were respectively identified as linolenic-oleic (main fragment ions at *m*/*z* 277; peak 31), linoleic-oleic (main fragment ions at *m*/*z* 279, corresponding to [M−H-C_18_H_34_O_2_]^−^; peak 32), and palmitic-oleic (displaying *m*/*z* 255, an ion associated with the deprotonated form of palmitic acid, C_16_H_31_O_2_^−^; peak 33) [22].

Loliolide (peak 3) was observed in positive ionization mode as a protonated ion with the formula C_11_H_17_O_3_^+^ (*m*/*z* 197). It exhibited two neutral losses: firstly, the loss of a water molecule, resulting in the ion with the formula C_11_H_15_O_2_^+^ (*m*/*z* 179), and, subsequently, the loss of a CO_2_ molecule, leading to the formation of an ion with the formula C_10_H_15_^+^ (*m*/*z* 135). Loliolide also displayed the loss of two water molecules, corresponding to the molecular formula C_11_H_13_O^+^ (*m*/*z* 161) [23].

Peaks 26 and 30, characterized by precursor ions at *m*/*z* 353 [M−H]^−^, were tentatively assigned as monolinolenin. The fragmentation pattern of this monoacylglycerol consistently exhibited a consistent loss of 92 Da, attributed to the cleavage of a glycerol moiety (C_3_H_8_O_3_), prominently manifesting as base peak ions at *m*/*z* 261. Notably, the successive removal of a water molecule from these intermediate ions gave rise to fragment ions at *m*/*z* 243 [24].

Phytosphingosine (peak 20) and dehydrosphingosine (peak 22) were both detected in the samples analyzed using a positive ionization mode. Phytosphingosine was linked to the precursor ion *m*/*z* 318 [M+H]^+^. The prevailing fragment associated with this compound arises from the loss of a water molecule, leading to [M+H-H_2_O]^+^. Subsequently, the loss of C_2_H_4_O gave rise to the fragment at *m*/*z* 256 [M+H-H_2_O-C_2_H_4_O]^+^. A sequence of consecutive cleavages then generated the ion corresponding to the molecular formula C_4_H_10_NO^+^ (*m*/*z* 88). Dehydrosphingosine, on the other hand, was associated with the precursor ion *m*/*z* 316 [M+H]^+^. Two successive losses of water molecules were observed, resulting in the formation of two fragments at *m*/*z* 298 [M+H-H_2_O]^+^ and *m*/*z* 280 [M+H-H_2_O-H_2_O]^+^, respectively. The fragment at *m*/*z* 60 was attributed to the ion with the molecular formula C_2_H_6_NO^+^. While identification was established for the majority of the compounds with support from the literature, both phytosphingosine and dehydrosphingosine were tentatively identified using the Compound Discoverer^TM^ software ver. 3.3.2.31.

Among all the elements detected in both negative and positive ionization modes, those that exhibited notably intense peaks in the chromatograms were attributed to the glycoalkaloid class. Tomatidine (peak 18), with a precursor ion [M+H]^+^ at *m*/*z* 416, showed a typical fragmentation pattern. This pattern was characterized by a water-loss ion at *m*/*z* 398 [M+H-H_2_O]^+^, along with two ions generated from the fragmentation of the ring attached to the spirosolane ring, resulting in hydrocarbon ions at *m*/*z* 273 and *m*/*z* 255 [25].

The precursor ion *m*/*z* 414 [M+H]^+^ was associated with solasodine (peak 17). This glycoalkaloid displayed a fragmentation pattern similar to the previously mentioned tomatidine: the predominant fragment observed was *m*/*z* 396, corresponding to the loss of a water moiety. In this case, fragmentation of the ring attached to the spirosolane ring generated two ions, *m*/*z* 271 and *m*/*z* 253 [26].

α-Tomatine (peak 10) was detected both in positive and negative ionization modes. The positive precursor ion at *m*/*z* 1034 [M+H]^+^ generated a *m*/*z* 1016 product ion, resulting from the loss of H_2_O [M+H-H_2_O]^+^. Ions at *m*/*z* 578 and *m*/*z* 416 corresponded to [Tomatidine+Gal+H]^+^ and [Tomatidine+H]^+^, respectively. These ions were formed by the removal of the Xyl-Glu(-Glu) moiety and the whole sugar chain from the tomatidine molecule [27]. In contrast to positive ionization, the negative precursor ion of the same molecule forms an adduct with formic acid, resulting in *m*/*z* 1078 [M+CH_2_O_2_-H]^−^ The loss of this formic acid adduct is evident from the main associated fragment mass (*m*/*z* 1032 [M−H-46]^−^). The mass spectrum also exhibits two additional fragments. The first one is attributed to the loss of a glucose unit, leading to an *m*/*z* 870 [M−H-46-162]^−^ fragment. In the second case, the subsequent loss of a xylose-glucose unit (132 + 162 Da) is observed, resulting in an *m*/*z* 576 [M−H-46-162-132-162]^−^ fragment [28].

Positive ionization mode analysis further revealed the presence of β2-tomatine (*m*/*z* 872 [M−H]^+^; peak 12). Its fragmentation pattern closely mirrored those observed for the previously discussed glycoalkaloids. Once again, the fragments resulted from the loss of a water molecule (*m*/*z* 854 [M+H-H_2_O]^+^), the complete removal of the sugar chain (*m*/*z* 416 [M+H-Xyl-Glu-Gal]^+^), and the generation of an *m*/*z* 255 ion [27].

LC/MS-MS analysis of the samples unveiled the presence of hydroxytomatine (peak 5) via a negative ionization mode, along with its stereoisomer neorickiioside A (peak 4), which displayed *m*/*z* 1050 [M+H]^+^ as the precursor ion. This led to two plausible losses: a water molecule at *m*/*z* 1032 [M+H-H_2_O]^+^ or the sugar chain (*m*/*z* 414). If both of these losses occurred simultaneously, the *m*/*z* 414 ion was formed [27].

Hydroxytomatine is found bound to formic acid, resulting in a precursor ion at *m*/*z* 1094 [M−H]^−^. Similar to other glycoalkaloids identified using a negative ionization mode, the primary fragments detected through the analysis correspond to ions generated by: formate loss at *m*/*z* 1048 [M−H-46]^−^, loss of a glucose unit at *m*/*z* 886 [M−H-Glu]^−^, and loss of a xylose-glucose unit at *m*/*z* 592 [M−H-Xyl-Glu(-Glu)]^−^ [29].

Dehydrotomatine (peak 8) showed a fragmentation pattern like that observed for α-tomatine when analyzed in negative ionization mode. The precursor ion *m*/*z* 1076 [M+CH_2_O_2_-H]^−^ once again represented an adduct formed as a result of binding with formic acid, and its loss was indicated by *m*/*z* 1030 [M−H-46]^−^. Subsequent deletions of the glucose moiety and the xylose-glucose unit led to the generation of ions at *m*/*z* 870 and *m*/*z* 574, respectively [29]. The protonated dehydrotomatine at *m*/*z* 1032 [M+H]^+^ generated an ion at *m*/*z* 576, corresponding to [M+H-Xyl-Glu(-Glu)-]^+^. Complete removal of the sugar chain and subsequent loss of a water molecule resulted in the generation of two additional ions at *m*/*z* 414 [Tomatidenol+H]^+^ and *m*/*z* 396 [Tomatidenol+H-H_2_O]^+^ [27].

**Table 1:** Complete list of tentatively identified compounds in tomato extracts.

Peak	Compounds	RT (min)	[M+H]^+^	MS/MS^+^	[M−H]^−^	MS/MS^−^	Chemical Formula	Error (ppm)	Class	DTE1	DTE2	PTE1	PTE2	DTA	PTA	Reference
1	Roseoside + FA	4.59			431.1919	385.1867 223.1334 161.0446	C_20_H_32_O_10_	1.67	Vomifoliol glucoside	×	×	×	×	×	-	[15]
2	Rutin	5.18	611.1609	303.0499	609.1459	301.0355	C_27_H_30_O_16_	0.99	Flavonoids	×	×	×	×	×	×	[15,16,30]
3	Loliolide	5.54	197.1172	179.106 135.1169 161.0961	197.1172	179.1066 135.1169 161.096	C_11_H_16_O_3_	0.22	Benzofurans	×	×	×	×	×	×	[16,23]
4	Neorickiioside A	5.6	1050.5461	1032.536 432.3483 414.3363	1050.5461	1032.536 432.3483 414.3363	C_50_H_83_O_22_N	−1.95	Dicarboxylic acid	×	×	×	×	×	×	[17,27]
5	Hydroxytomatine + FA	5.63			1094.5398	1048.5343 886.4812 592.3854	C_51_H_85_O_24_N	1.84	Glycoalkaloids	×	×	×	×	-	-	[29]
6	Azelaic acid	5.7			187.0967	125.0959 169.086 97.0646	C_9_H_16_O_4_	1.04	Dicarboxylic acid	×	×	×	×	×	-	[17]
7	4-Oxododecanedioic acid	5.78			243.1234	225.1127 99.0074 181.1224	C_12_H_20_O_5_	2.56	Fatty acyl	×	×	×	×	×	-	[18]
8	Dehydrotomatine	6.21	1032.5363	414.3366 576.3876 396.3253	1032.5347	414.3359 396.3260 576.3884	C_50_H_81_O_21_N	−0.5	Glycoalkaloids	×	×	×	×	×	×	[18,27]
9	Dehydrotomatine + FA	6.22			1076.5283	1030.5236 868.47 574.3736	C_51_H_83_O_23_N	2.16	Glycoalkaloids	×	×	×	×	×	-	[29]
10	α-Tomatine	6.32	1034.5515	416.352 1016.5422 578.4045	1034.5504	416.3521 578.4056 1016.5408	C_50_H_83_O_21_N	−0.28	Glycoalkaloids	×	×	×	×	×	×	[27,29]
11	α-Tomatine FA	6.32			1078.5425	1032.5378 870.4856 900.4962	C_51_H_85_O_23_N	1.34	Glycoalkaloids	×	×	×	×	×	-	[28]
12	β2-Tomatine	6.42	872.4991	416.3500 255.2100 854.4854	872.4996	416.3516 255.2107 854.4893	C_44_H_73_NO_16_	−0.52	Glycoalkaloids	×	×	×	×	×	×	[27,28]
13	Apo-13-zeaxanthinone	6.9	275.2004	133.1013 147.1169 257.1898	275.1989	257.1896 133.1013 119.0857	C_18_H_26_O_2_	0.35	Sesquiterpenoids	×	×	×	×	×	×	[20,21,31]/HMDB
14	Malyngic acid	6.91			327.2174	211.1332 229.1439 171.1015	C_18_H_32_O_5_	0.84	Fatty acid	×	×	×	×	×	-	[20,21]
15	Trihydroxy-10-trans-octadecenoic acid	7.2			329.2331	211.1334 229.114 171.1015	C_18_H_34_O_5_	0.96	Fatty acid	×	×	×	×	×	-	[32]
16	Trihydroxy-10-trans-octadecenoic acid *isomer*	7.57			329.2332	211.1334 229.1440 171.1015	C_18_H_34_O_5_	0.87	Fatty acid	×	×	×	×	-	-	[32]
17	Solasodine	7.7	414.3364	396.3267 271.2056 253.1957			C_27_H_43_O_2_N	−0.56	Alkaloids	×	×	×	×	-	-	[26]
18	Tomatidine	7.9	416.3513	398.3410 273.2209 114.0919	416.3515	273.2205 398.3425 255.2114	C_27_H_45_O_2_N	−0.56	Glycoalkaloids	×	×	×	×	×	×	[25,32]
19	16-hydroxy-9-oxo-10E,12E,14E-octadecatrienoic acid	7.92			307.1914	235.1336 185.1174 121.0646	C_18_H_28_O_4_	2.73	Fatty acid	×	×	×	×	-	-	[19]
20	Phytosphingosine	8.02	318.3002	256.2634 300.2904 88.0764	318.2997	256.2632 99.9347 88.0761	C_18_H_39_O_3_N	−0.22	Sphingoid	×	×	×	×	×	×	[31,33,34]/HMDB
21	Phytuberin	8.17			293.1758	236.1051 221.1542 71.0125	C_17_H_26_O_4_	4.40	Sesquiterpenoid	-	-	-	-	×	-	[33,34]
22	Dehydrophytosphingosine	8.43	316.2843	280.263 360.0451 298.3274			C_18_H_37_O_3_N	0.05	Sphingoid	×	×	×	×	-	-	[31]/HMDB
23	Palmitoleic-linolenic glucoside	8.66			721.3651	397.1348 277.2171 415.1456	C_34_H_58_O_16_	1.05	Fatty acid	×	×	×	×	-	-	[22]
24	Hexadecatrienoic acid	8.71			249.1857	205.1953 59.0125 231.1751	C_16_H_26_O_2_	0.76	Fatty acid	×	×	×	×	-	-	HMDB
25	Wilfoside D FA	8.75			1107.5336	1061.5292 899.4764 605.3807	C_56_H_84_O_22_	−3.14	Steroidal glycosides	-	-	-	-	×	×	[35]
26	Monolinolenin	8.79	353.2685	261.2214 95.0860 243.2109			C_21_H_36_O_4_	0.03	Fatty Acyls	×	×	×	×	-	-	[24]
27	Palmitoleic-linolenic glucoside *isomer*	8.81			721.3468	397.1346 277.217 415.1454	C_34_H_58_O_16_	0.79	Fatty acid	×	×	×	×	-	-	[22]
28	9-hydroxy-10E,12Z,15Z-octadecatrienoic acid	9.31			293.2122	275.2015 235.1700 171.1017	C_18_H_30_O_3_	3.54	Fatty acid	×	×	×	×	-	-	[19]
29	Stearidonic acid	9.36	277.2161	135.1169 121.1013 93.0702			C_19_H_28_O_2_	−0.14	Fatty acid	×	×	×	×	-	-	[31]
30	Monolinolenin *isomer*	9.49	353.2683	261.2213 243.2106 95.0859			C_21_H_36_O_4_	0.02	Fatty Acyls	×	×	×	×	-	-	[24]
31	Linolenic-oleic	9.5			559.3119	277.217 253.0926 513.3065	C_28_H_48_O_11_	0.31	Fatty acid	×	×	×	×	-	-	[22]
32	Linoleic-oleic	9.99			561.3275	279.2327 253.0926 515.3224	C_28_H_50_O_11_	1.24	Fatty acid	×	×	×	×	-	-	[22]
33	Palmitic-oleic	10.37			537.3284	255.2327 491.323 235.0819	C_26_H_50_O_11_	2.66	Fatty acid	×	×	×	×	-	-	[22]
34	16-hydroxyhexadecanoic acid	11.11			271.2278	225.217	C_16_H_32_O_3_	3.62	Fatty acid	×	×	×	×	-	-	[36]

FA = formic acid; × and - indicate the presence or absence of compounds in the vegetable extracts, respectively; HMDB: Human Metabolome Database.

#### Quantification of α-TM and TD in Tomato Extracts

As reported in the literature [3,37,38], the health properties of tomato leaves extracts were due to the high alkaloids content (α-TM and TD). Therefore, the quantification of both compounds was also carried out (Table S1).

### 2.2. In Vitro Assay: Percentage of Cell Viability

Several studies have demonstrated that the consumption of tomato is useful for the prevention of many diseases, including cancer. In fact, the tomato contains several natural antioxidants such as lycopene, the steroidal alkaloid TD, and its glycoside α-TM. In particular, it was observed in vitro and in vivo models that α-TM and/or TD possess strong anticancer activity [37,38].

**Figure 1:**
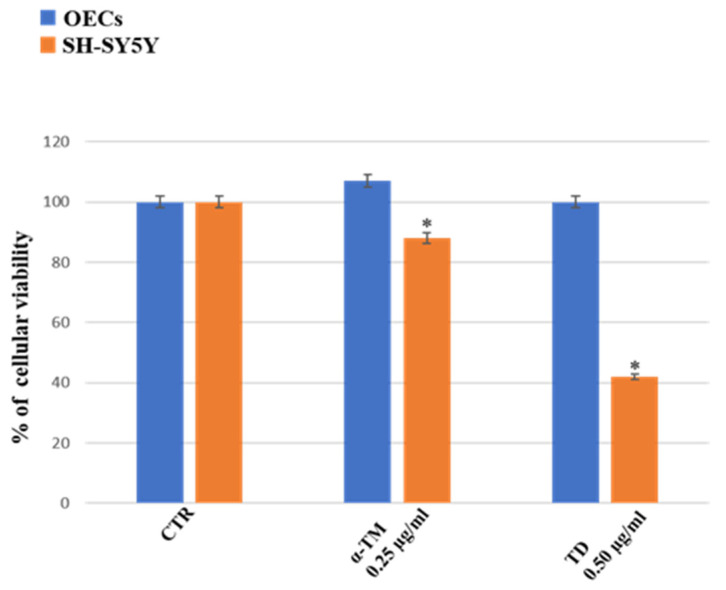
Percentage of OECs (blue) and SH-SY5Y (orange) viability. Untreated cell (CTR), α-TM at 0.25 µg/mL, and TD at 0.50 µg/mL for 24 h. * *p* < 0.05 difference vs. CTR.

Our study, performed using commercial steroid alkaloids (α-TM and TD), free and loaded into SLNs, was able to reduce the percentage of cell viability on human neuroblastoma cell lines (SH-SY5Y) assessed at different concentrations and time of exposure [39]. In addition, we evaluated its effect on OECs, using them as control of health cells [39]. To monitor the percentage of cell viability on OECs and SH-SY5Y cell cultures in the absence and in the presence of PTE1, PTA, DTE1, DTE2, and DTA, MTT was performed. Furthermore, we compared the data obtained with α-TM (0.25 µg/mL) and TD (0.50 µg/mL), chosen at the optimal concentration previously observed (Figure 1).

**Figure 2:**
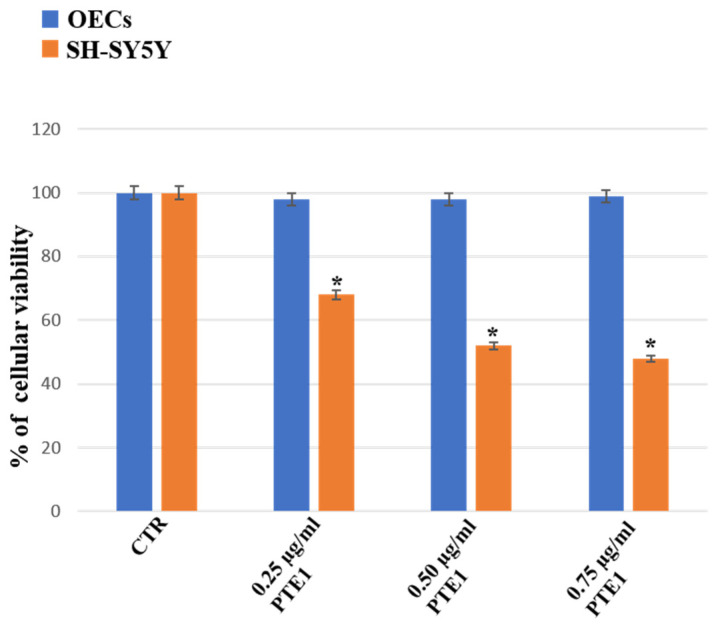
Percentage of cell viability on OEC (blue) and SH-SY5Y (orange) cell cultures in the absence (CTR) and in the presence of different concentrations of 0.25 µg/mL, 0.50 µg/mL, or 0.75 µg/mL of PTE1 for 24 h. Data represent the mean ± S.D. of five separated experiments performed in triplicate. * *p* < 0.05 significant differences vs. CTR.

Figure 2 shows the treatment of OEC and SH-SY5Y cell cultures at different concentrations of PTE1 (0.25 µg/mL, 0.50 µg/mL, 0.75 µg/mL) for 24 h. We found that PTE1 did not induce any significant change in the percentage of OEC viability at all concentrations when compared with the untreated ones used as a control. In contrast, a significant decrease in the percentage of SH-SY5Y viability when exposed to the concentrations of PTE1 was observed when compared with the control. The effect was particularly evident in SH-SY5Y cultures treated with PTE1, especially at the concentration of 0.50 µg/mL and 0.75 µg/mL, when compared to the control.

**Figure 3:**
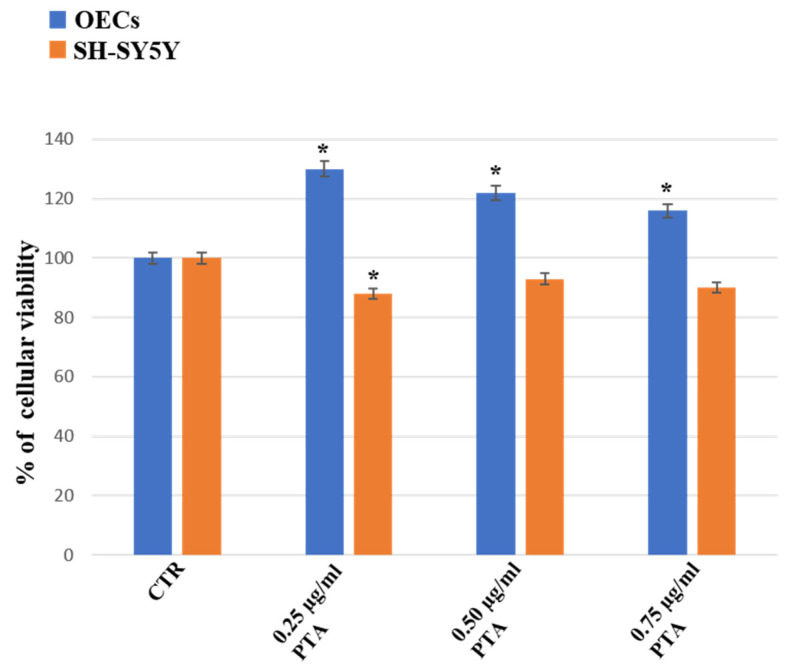
Percentage of cell viability on OEC (blue) and SH-SY5Y (orange) cell cultures in the absence (CTR) and in the presence of different concentrations of 0.25 µg/mL, 0.50 µg/mL, or 0.75 µg/mL of PTA for 24 h. Data represent the mean ± S.D. of five separated experiments performed in triplicate. * *p* < 0.05 significant differences vs. CTR.

The treatment of OECs for 24 h with PTA at the concentration of 0.25 µg/mL, 0.50 µg/mL, and 0.75 µg/mL induced a significant increase in the percentage of OEC viability when compared with the control (Figure 3). In SH-SY5Y cell cultures exposed at the same concentrations of PTA, a slight significant decrease in the percentage of cell viability was found when compared with the respective control and the OECs exposed at the same concentration of the extract (Figure 3).

**Figure 4:**
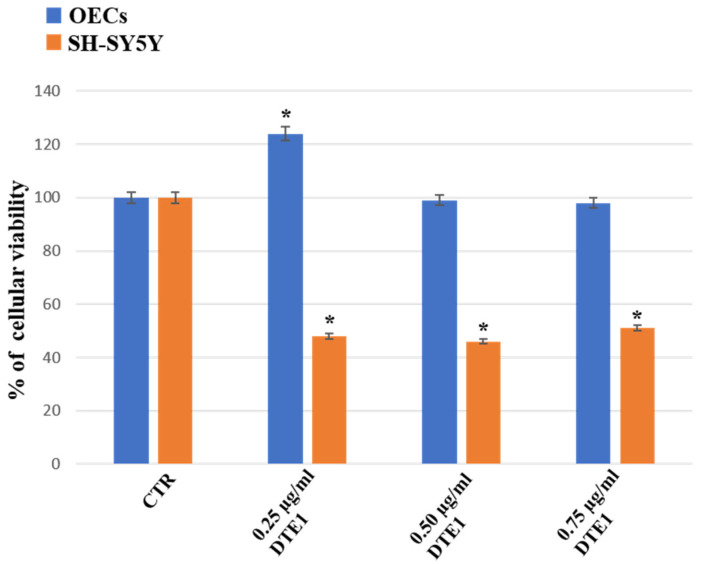
Percentage of cell viability on OEC (blue) and SH-SY5Y (orange) cell cultures in the absence (CTR) and in the presence of different concentrations of 0.25 µg/mL, 0.50 µg/mL, or 0.75 µg/mL of DTE1 for 24 h. Data represent the mean ± S.D. of five separated experiments performed in triplicate. * *p* < 0.05 significant differences vs. CTR.

Figure 4 highlights the effect of the treatment with 0.25 µg/mL, 0.50 µg/mL, or 0.75 µg/mL of DTE1 on OEC and SH-SY5Y cell cultures. A significant enhancement in the percentage of cell viability on OECs exposed to 0.25 µg/mL of DTE1 was found when compared with the respective control. No significant change was observed on OECs treated with 0.50 µg/mL or 0.75 µg/mL of DTE1. The exposure of SH-SY5Y cell cultures to all the concentrations of the extract was able to induce a significant decrease in the percentage of cell viability when compared with the control and OECs treated with the DTE1 at the same concentration (Figure 4).

**Figure 5:**
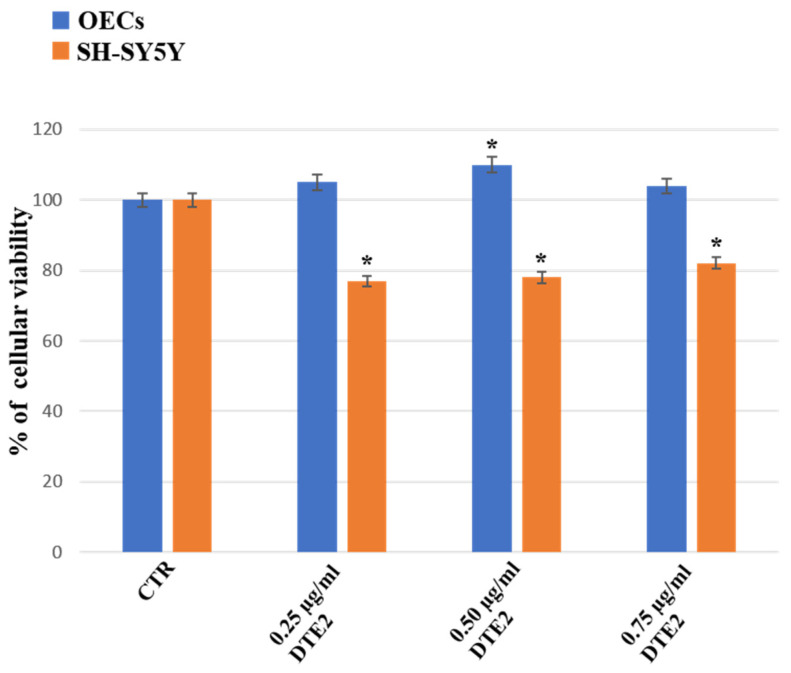
Percentage of cell viability on OEC (blue) and SH-SY5Y (orange) cell cultures in the absence (CTR) and in the presence of different concentrations of 0.25 µg/mL, 0.50 µg/mL, or 0.075 µg/mL of DTE2 for 24 h. Data represent the mean ± S.D. of five separated experiments performed in triplicate. * *p* < 0.05 significant differences vs. CTR.

Figure 5 reports the effect of the treatment for 24 h for both OEC and SH-SY5Y cell cultures with DTE2 at different concentrations (0.25 µg/mL, 0.50 µg/mL, 0.75 µg/mL) on the percentage of cell viability. The treatment of OECs with 0.25 µg/mL or 0.75 µg/mL of DTE2 did not lead to any significant change in the percentage of cell viability when compared with the control. A slight but significant enhancement in OECs exposed to 0.50 µg/mL of DTE2 was observed. In SH-SY5Y DTE2-treated cells, a significant decrease in the percentage of cell viability at all concentrations was found when compared with the control and OEC treated ones.

The treatment with DTA was performed at concentrations of 0.025 µg/mL, 0.05 µg/mL, 0.075 µg/mL, or 0.01 µg/mL, since concentrations 0.25 µg/mL, 0.50 µg/mL, and 0.75 µg/mL used for the other extracts did not induce any significant changes in OEC or SH-SY5Y cell cultures when compared with the respective control.

**Figure 6:**
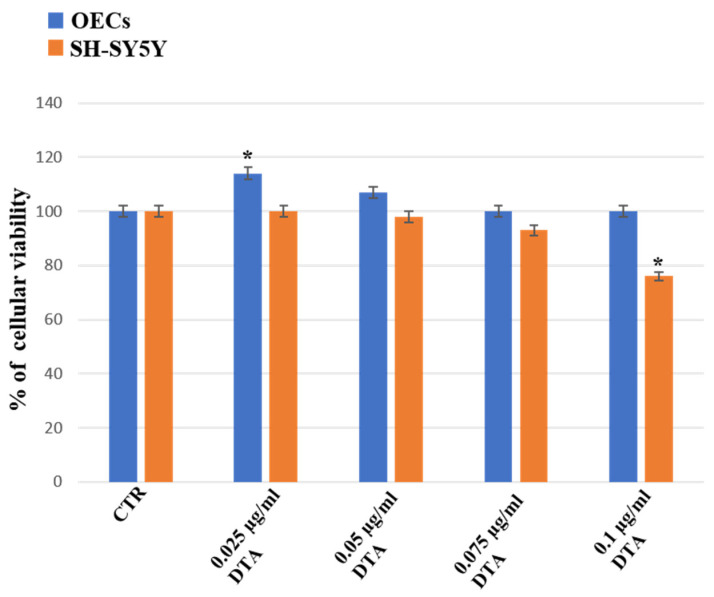
Percentage of cell viability on OEC (blue) and SH-SY5Y (orange) cell cultures in the absence (CTR) and in the presence of different concentrations of 0.025 µg/mL, 0.05 µg/mL, 0.075 µg/mL, or 0.01 µg/mL of DTA for 24 h. Data represent the mean ± S.D. of five separated experiments performed in triplicate. * *p* < 0.05 significant differences vs. CTR.

Figure 6 shows that the treatment for 24 h of OECs with DTA at the concentration of 0.025 µg/mL, 0.05 µg/mL, 0.075 µg/mL, or 0.01 µg/mL did not induce a significant change in the percentage of cell viability when compared with the control. A slight increase after treatment at 0.025 µg/mL DTA in the percentage of cell viability was observed when compared to the control and OEC treated ones. No significant change in cell viability was observed when the SH-SY5Y cell cultures were exposed at 0.025 µg/mL and 0.050 µg/mL of DTA when compared with the control. A significant decrease in percentage of cell viability in SH-SY5Y was observed when the cells were treated with DTA at concentrations of 0.075 µg/mL and 0.1 µg/mL when compared with the control and OEC treated ones. We chose OEC cell line as a control, for their proliferative activity and stem cell characteristics [40], and SH-SY5Y neuroblastoma cells for evaluating the neuroprotective effect of the tomato steroidal alkaloids [37].

## 3. Methods and Materials

### 3.1. Materials

Mice pups were provided by Envigo RMS s.r.l., San Pietro al Natisone (UD), Italy, stock: C57BL6. SH-SY5Y human neuroblastoma cell line was purchased from Cell Bank Interlab Cell Line Collection (ICLC) (Genova, Italy). Antibiotics, trypsin, non-essential amino acids, phosphate buffer saline solution (PBS), cytosine arabinoside, health inactivated fetal bovine serum, and modified Eagle medium (MEM) with 2 mM GlutaMAX were obtained from GIBCO (Milan, Italy). Ham’s F12, [3(4,5-dimethyl-thiazol-2-yl)2,5-diphenyl-tetrazolium bromide), MTT] and other chemicals were purchased from Merck (Milan, Italy).

### 3.2. Extraction

Tomato leaves of DT and PT were harvested in September 2022 from a glasshouse farm located in Pachino (Syracuse, Italy). The fresh plant fractions were dried at 60 °C for 48 h and subsequently pulverized by a mechanical mill; the obtained powders were used for aqueous and ethanolic extractions.

The aqueous extract of each cultivar (DTA and PTA) was prepared by adding 10 g of powder in 100 mL of deionized water for 48 h at room temperature [41]. The resulting mixture was filtered using paper filter, frozen, and freeze-dried (Lio 5P-Pascal SRL, Milan, Italy). Concerning ethanolic extract, 35 g of each variety were macerated in a mixture of ethanol and acetic acid (95:5 *v*/*v*) for 72 h at room temperature [3]. The supernatant was collected (first extraction: DTE1 and PTE1), filtered, concentrated under vacuum, and freeze-dried (Lio 5P-Pascal SRL, Milan, Italy). In order to exhaust the plant matrices, the residue was further extracted with the acid-ethanol mixture (second extraction: DTE2 and PTE2) and subjected to the same experimental procedure.

### 3.3. LC-MS/MS Conditions

UHPLC-HRMS/MS analysis was performed on a Thermo Ultimate RS 3000 coupled online to a Q-Exactive hybrid quadrupole Orbitrap mass spectrometer (Thermo Fisher Scientific, Bremen, Germany) equipped with a heated electrospray ionization probe (HESI II). The separation was performed in reversed phase mode, with a Luna Omega Polar C18 (100 × 2.1 mm × 1.6 μm) (Phenomenex, Bologna, Italy). The column temperature was set at 40 °C and the flow rate was 0.3 mL min^−1^. The mobile phase was (A) H_2_O with 0.1% HCOOH (*v*/*v*) and (B) ACN with 0.1% HCOOH (*v*/*v*). The following gradient was employed: 0.01–10.00 min, 5–95% B; 10.01–12.00 min, isocratic to 95% B; 12.01–13.00 min, 5% B; then five minutes for column re-equilibration. Five μL were injected.

The ESI was operated in positive and negative mode. The MS was calibrated by Thermo calmix Pierce™ calibration solutions in both polarities. Full MS (150–1500 *m*/*z*) and data-dependent MS/MS were performed at a resolution of 35,000 and 17,500 FWHM, respectively; normalized collision energy (NCE) values of 15, 20, and 25 were used. Source parameters were sheath gas pressure, 50 arbitrary units; auxiliary gas flow, 13 arbitrary units; spray voltage, +3.5 kV, −2.8 kV; capillary temperature, 320 °C; auxiliary gas heater temperature, 350 °C.

The identification of investigated analytes was carried out by comparing their retention times and MS/MS data with those present in the literature. Data analysis and processing were performed using FreeStyle™ 1.8 SP2 and Compound Discoverer 3.3.1 (Thermo Fisher Scientific, Bremen, Germany). The following online databases were also consulted: Phenol-Explorer (http://phenol-explorer.eu/), PubChem (https://pubchem.ncbi.nlm.nih.gov), ChemSpider (http://www.chemspider.com), SciFinder Scholar (https://scifinder.cas.org), TOMATOMET (http://metabolites.in/tomato-fruits), HMDB (https://hmdb.ca/).

### 3.4. In Vitro Assay

#### 3.4.1. Primary OEC Cultures

OECs were obtained from mice pup (P2) olfactory bulbs and processed according to the method by Pellitteri et al. [42]. Collagenase and trypsin were used to digest the tissue. Subsequently, DMEM was added with 10% FBS to block trypsinization. Cell suspension was then placed in 75 cm^2^ flasks and fed with DMEM/FBS added with penicillin/streptomycin (50 U/mL). After 24 h, cytosine arabinoside (10^−5^ M), an agent used to reduce the number of dividing fibroblasts, was added. Subsequently, the cell cultures were further purified using the method of Chuah and Teague [43]. Finally, OECs were incubated at 37 °C in an environment with humidified air and CO_2_ (95–5%). The medium culture was changed 2 times per week. The cell cultures were characterized morphologically through immunocytochemical procedures using S-100/p75, specific markers for OECs [44].

#### 3.4.2. SH-SY5Y Cell Line Cultures

SH-SY5Y cell line cultures were obtained through cell suspension in complete culture medium containing Ham’s F12 and MEM (1:1), 10% (*v*/*v*) FBS, 2 mM GlutaMAX, 50 mg/mL penicillin/streptomycin (50 U/mL). The cell suspension was plated in 75 cm^2^ flasks at a final density of 2 × 10^6^ cells and incubated at 37 °C in humidified air and CO_2_ (95–5%). The culture medium was changed every 2–3 days. When the cell cultures reached approximately 80–85% confluence, they were subcultured at a density ratio of 1:4 and incubated at 37 °C in a humidified atmosphere containing CO_2_ (95–5%).

#### 3.4.3. Treatment of Cells

Primary OECs and SH-SY5Y cell line cultures were exposed for 24 h to the following treatments: a group of cell cultures was treated with PTE1/PTA/DTE1/DTE2 extract at different concentrations (0.25 µg/mL, 0.50 µg/mL, or 0.75 µg/mL); another group was treated with DTA (0.025 µg/mL, 0.05 µg/mL, 0.075 µg/mL, or 0.01 µg/mL); a group of cells was treated with a corresponding volume of PBS (final concentration 0.01% *v*/*v*), used as a control (CTR); another group of cells was treated with α-tomatine (0.25 µg/mL) for 24 h, utilized as commercial steroidal alkaloid present in the tomato.

#### 3.4.4. MTT Assay

To monitor cell viability, an MTT test was used [45]. Briefly, cells were set up 0.5 × 10^4^ cells per well of a 96-multiwell, flat-bottomed, 200-µL microplate and maintained at 37 °C in humidified air mixture and CO_2_ (95–5%). At the end of treatment time, 20 µL of 0.5% MTT in (pH 7.4) PBS was added to each microwell. After 2 h, the supernatant was removed and replaced with 100 µL of DMSO. The optical density of each one was measured with a microplate spectrophotometer reader (Titertek Multiskan; Flow Laboratories, Helsinki, Finland) at λ = 570 nm. Data were expressed as a percentage of PBS (control), as taken as 100%, to normalize the values.

#### 3.4.5. Statistical Analysis

To assess the significant differences among groups, data were analyzed through one-way analysis of variance (ANOVA) followed by a post hoc Holm–Sidak. Results were reported as mean ± SD of four separated experiments performed in triplicate, and differences between groups were considered to be significant at * *p* < 0.05.

## 4. Conclusions

An in-depth study of different extracts obtained from the leaves of tomato cultivars, including “*Datterino*” tomato (DT) and “*Piccadilly*” tomato (PT), has provided valuable insights into the complex phytochemical composition. LC/MS-MS analyses performed in both positive and negative ionization modes allowed us to tentatively identify a wide range of compounds belonging to the classes of alkaloids, flavonoids, fatty acids, lipids, and terpenes. Furthermore, the potential anticancer activity of different extracts was evaluated in vitro by MTT assay. In particular, the percentage of cell viability was assessed on OECs, a particular glial cell type of the olfactory system, and on SH-SY5Y, a neuroblastoma cell line. All extracts did not lead to any significant change in the percentage of cell viability on OECs when compared with the control. Instead, in SH-SY5Y we observed a significant decrease in the percentage of cell viability, confirming their potential anticancer activity. This was more evident for the ethanolic extracts due to the high alkaloids content (Table S1). Therefore, the results highlight the nutraceutical potential of tomato leaves as a valuable source of bioactive compounds, suitable for various applications in the food and pharmaceutical industries.

## Data Availability

The datasets used and/or analyzed during the current study are available from the corresponding author on reasonable request.

## Supplementary Materials

The following supporting information can be downloaded at https://www.mdpi.com/article/10.3390/ijms242316915/s1.
