# Supplementary material for: Steroidal Alkaloids from Food Waste of Tomato Processing Inhibit Neuroblastoma Cell Viability
Source: Int J Mol Sci. 2023 Nov 29;24(23):16915. doi: 10.3390/ijms242316915 (PMC10706926; doi:10.3390/ijms242316915)
Supplement: Supplementary file 1 [file ijms-24-16915-s001.zip › ijms-2680592-supplementary.pdf]

Supporting Information

Steroidal alkaloids from food waste of tomato processing inhibit neuroblastoma cell viability

A

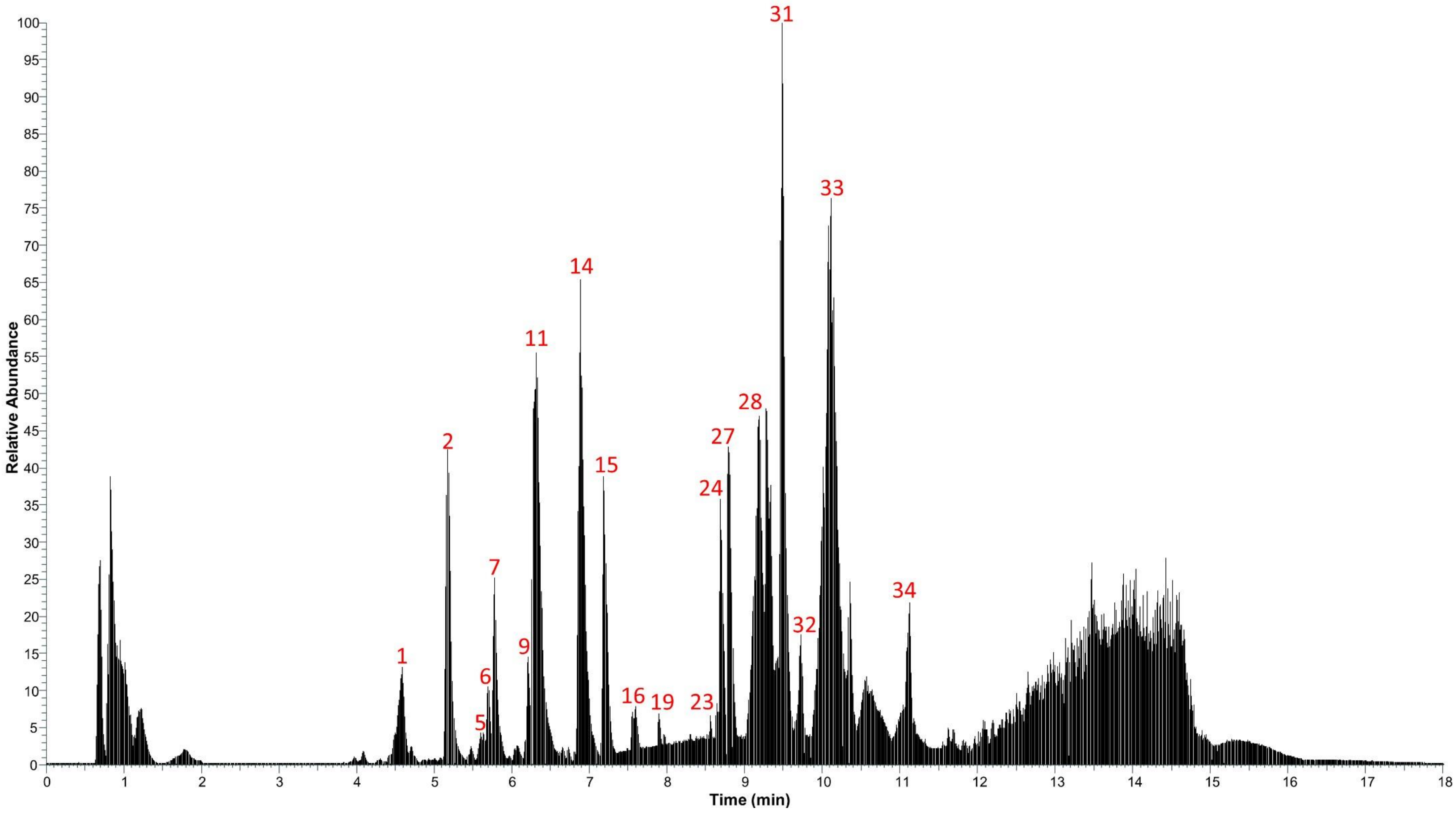

B

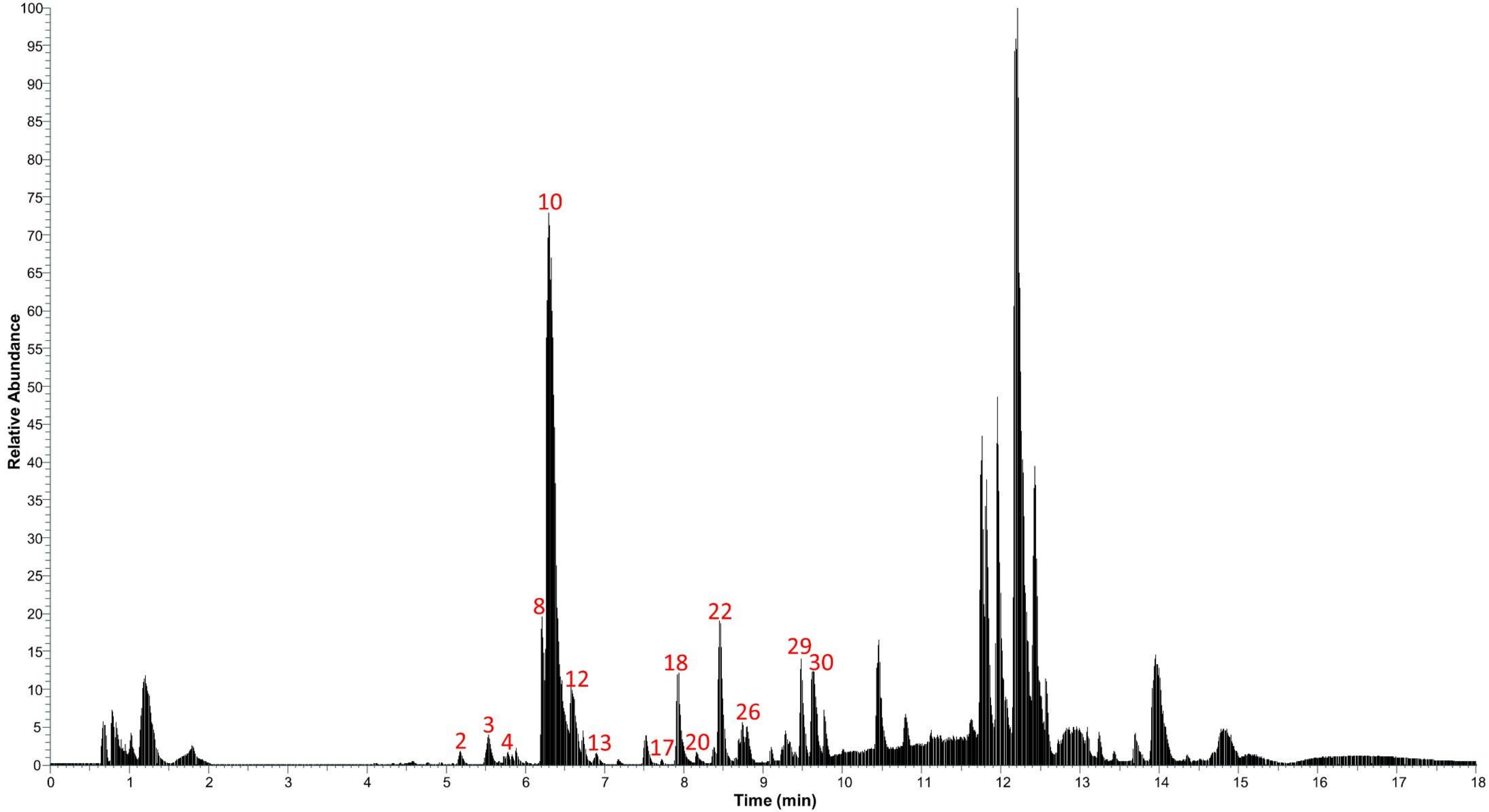

Figure S1. Representative total ion chromatograms (TICs) of tomato ethanolic extract analyzed in negative (A) and positive (B) ionization modes.

A

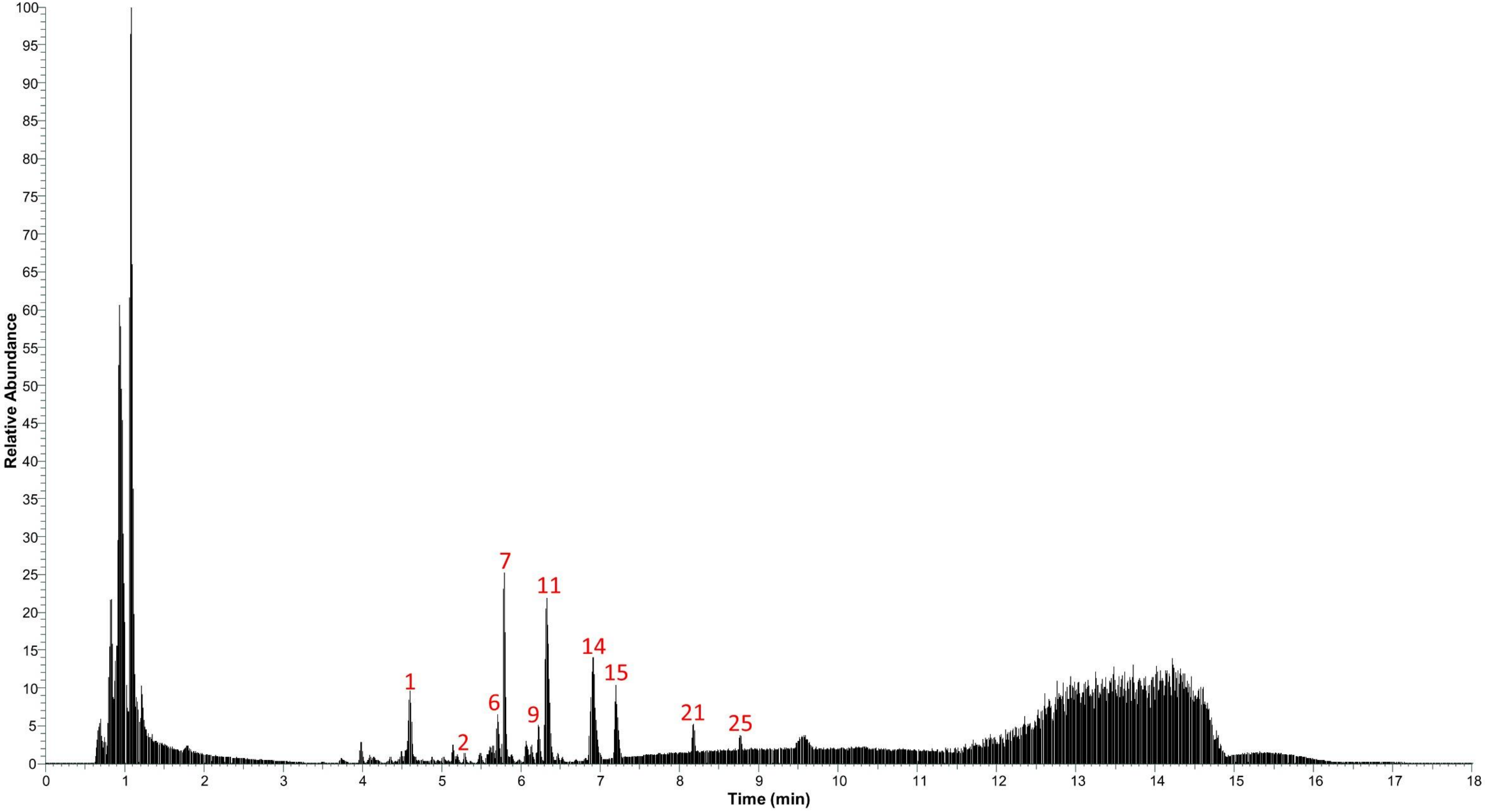

Relative Abundance
100
95
90
85
80
75
70
65
60
55
50
45
40
35
30
25
20
15
10
5
0
1
2
6
7
9
11
14
15
21
25
0
1
2
3
4
5
6
7
8
9
10
11
12
13
14
15
16
17
18
Time (min)

B

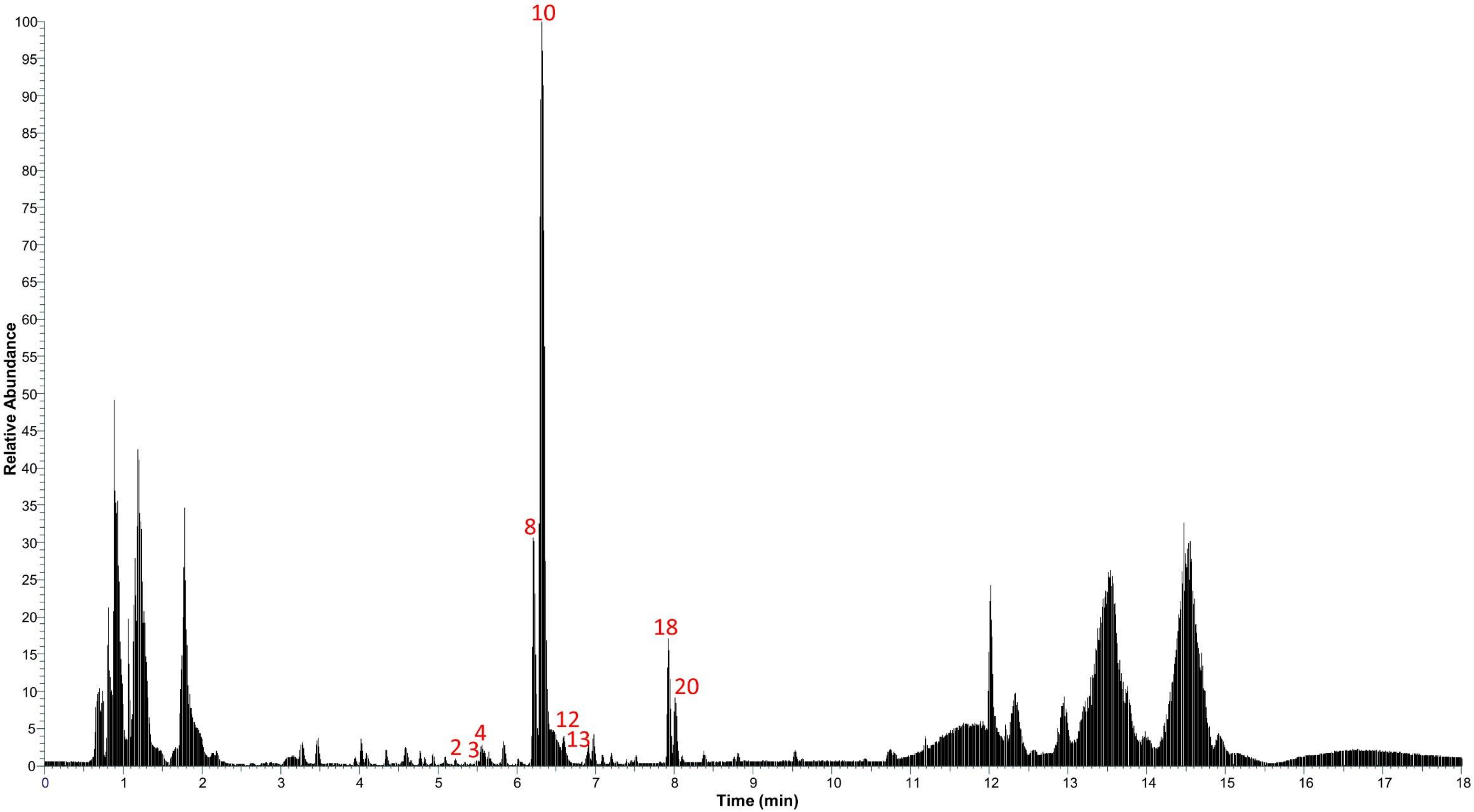

Figure S2. Representative TICs of tomato aqueous extract analyzed in negative (A) and positive (B) ionization modes.

Table S1. Quantification of α-TM and TD in tomato extracts.

Extract	α-TM<br>µg g-1<br>(Analyte/dry extract)	TD<br>µg g-1<br>(Analyte/dry extract)
DTE1	47.9 ± 0.2	12.80 ± 0.11
DTE2	51.1 ± 0.4	10.69 ± 0.24
DTA	3.6 ± 0.2	<LOQ
PTE1	136.7 ± 2.1	24.19 ± 0.19
PTE2	95.3 ± 1.8	11.07 ± 0.59
PTA	6.7 ± 0.01	<LOQ
